# Innate Immune Interactions between *Bacillus anthracis* and Host Neutrophils

**DOI:** 10.3389/fcimb.2018.00002

**Published:** 2018-01-22

**Authors:** Janet Z. Liu, Syed R. Ali, Ethan Bier, Victor Nizet

**Affiliations:** ^1^Division of Host-Microbe Systems and Therapeutics, Department of Pediatrics, University of California, San Diego, San Diego, CA, United States; ^2^Section of Cell and Developmental Biology, Division of Biological Sciences, University of California, San Diego, San Diego, CA, United States; ^3^Skaggs School of Pharmacy and Pharmaceutical Sciences, University of California, San Diego, San Diego, CA, United States

**Keywords:** *Bacillus anthracis*, anthrax, neutrophils, lethal toxin, edema toxin, innate immunity, neutrophil extracellular traps, host-pathogen interactions

## Abstract

*Bacillus anthracis*, the causative agent of anthrax, has been a focus of study in host-pathogen dynamics since the nineteenth century. While the interaction between anthrax and host macrophages has been extensively modeled, comparatively little is known about the effect of anthrax on the immune function of neutrophils, a key frontline effector of innate immune defense. Here we showed that depletion of neutrophils significantly enhanced mortality in a systemic model of anthrax infection in mice. *Ex vivo*, we found that freshly isolated human neutrophils can rapidly kill anthrax, with specific inhibitor studies showing that phagocytosis and reactive oxygen species (ROS) generation contribute to this efficient bacterial clearance. Anthrax toxins, comprising lethal toxin (LT) and edema toxin (ET), are known to have major roles in *B. anthracis* macrophage resistance and systemic toxicity. Employing isogenic wild-type and mutant toxin-deficient *B. anthracis* strains, we show that despite previous studies that reported inhibition of neutrophil function by purified LT or ET, endogenous production of these toxins by live vegetative *B. anthracis* failed to alter key neutrophil functions. The lack of alteration in neutrophil function is accompanied by rapid killing of *B. anthracis* by neutrophils, regardless of the bacteria's expression of anthrax toxins. Lastly, our study demonstrates for the first time that anthrax induced neutrophil extracellular trap (NET) formation.

## Introduction

*Bacillus anthracis* is the causative agent of anthrax, and can infect hosts via respiratory, cutaneous, and gastrointestinal routes. *B. anthracis* produces a number of virulence factors, the most extensively studied of which is the tripartite anthrax toxin. Anthrax toxin is composed of the receptor binding protective antigen (PA), the metalloprotease lethal factor (LF) and the calmodulin-dependent adenylate cyclase edema factor (EF). The combination of PA and LF generates lethal toxin (LT), which cleaves and inactivates members of the mitogen-activated protein kinase kinase (MEK) family and inflammasome sensor protein NLRP1 in host cells (Duesbery et al., 1998; Levinsohn et al., 2012). PA also interacts with EF to create edema toxin (ET), which raises intracellular cAMP levels to promote cation efflux and impair endosomal recycling, leading to edema and disruption of barrier function (Leppla, 1982; Guichard et al., 2010). During infection, PA binds two cellular receptors, capillary morphogenesis protein-2 (CMG2) and tumor endothelium marker-8 (TEM8), leading to endocytosis of LF and EF into the cell (Bradley et al., 2001; Scobie et al., 2003). Once in the cytosol, LF and EF can interfere with critical cellular processes and promote accelerated cell death.

Neutrophils are key front line innate immune cells for control and clearance of bacterial pathogens. Neutrophils migrate to the site of infection where they can deploy reactive oxygen species (ROS), antimicrobial peptides and proteases, and neutrophil extracellular traps (NETs) to immobilize and kill bacteria. Their importance is highlighted in neutropenic patients, who are markedly more susceptible to bacterial and fungal infections and associated mortality (Bodey, 1986; Rolston, 2004). In the context of anthrax infection, neutrophils can kill the vegetative and spore form of the bacterium, and neutropenic mice are more susceptible to aerosol spore challenge with *B. anthracis* Ames (Mayer-Scholl et al., 2005; Cote et al., 2006). Knockout mice with myeloid-specific deletion of anthrax receptor CMG2 are more resistant than wild-type (WT) animals to mortality induced by *B. anthracis* spore challenge; however, this resistance is abolished when neutrophils are depleted, suggesting that neutrophils play an important role in controlling infection (Liu et al., 2010). While such studies indicate that neutrophils have an important role in controlling anthrax infection, the mechanistic interactions between neutrophils and metabolically active vegetative *B. anthracis* during infection remain unclear. Treatment of neutrophils with purified LT or ET can affect specific neutrophil functions, including chemotaxis, ROS production, and phagocytosis (O'brien et al., 1985; Wade et al., 1985; Crawford et al., 2006; Szarowicz et al., 2009; Weiner et al., 2014). However, some of the observed phenotypes have been contradictory, and the use of purified toxin alone cannot recapitulate the true scope of interaction between neutrophils with live anthrax bacteria. In this study, our aim is to probe the mechanisms through which neutrophils contribute to the control of anthrax infection, and the effect of anthrax toxin during neutrophil infection with vegetative *B. anthracis*.

## Materials and methods

### Bacterial strains and culture conditions

*B. anthracis* Sterne 7702 is a Biosafety Level-2 pathogen which we study under protocols reviewed and approved by the UC San Diego Institutional Biosafety Committee. WT *B. anthracis* Sterne 7702 and isogenic mutant strains lacking expression of the edema factor-encoding *cya* gene (ΔEF), lethal factor-encoding *lef* gene (ΔLF), or the protective antigen gene *pag* (Δ*pagA*) were propagated with continuous agitation in brain-heart infusion (BHI) medium (BD Biosciences) at 37°C. For phagocytosis assays, GFP was expressed in *B. anthracis* via electroportation of the pDCerm-GFP plasmid, in which the e-*gfp* gene is constitutively expressed behind *tet, cat* and *erm* promoters (Ly et al., 2014). For experiments, *B. anthracis* were grown in BHI media alone, or BHI + 5 μg/ml erythromycin to maintain plasmid pDCerm-GFP expression, to early log phase corresponding to an optical density at 600 nm (OD_600nm_) = 0.4, spun down, and washed with assay specific media.

### Neutrophil isolation

Human blood was collected from healthy volunteers with written informed consent according to protocol for simple phlebotomy approved by the UCSD IRB/Human Research Protection Program. Neutrophils were separated from whole blood as previously described (Von Köckritz-Blickwede et al., 2010); briefly, whole blood was layered over Polymorphprep (Axis-Shield) and spun in a centrifuge for 30 min at 512 × g without brakes. The polymorphonuclear cell layer was isolated, and erythrocytes lysed using sterile molecular grade water. After erythrocyte lysis, neutrophils were washed with sterile PBS and cell number determined by trypan blue (Invitrogen) staining on a hemocytometer.

### Mouse infection and survival studies

All studies were performed under a protocol approved by the UC San Diego Institutional Animal Care and Use Committee. Eight to Twelve weeks old female C57BL/6 mice (Charles River Laboratories) were injected intraperitoneally with 2–5 × 10^5^ colony forming units (CFU) of *B. anthracis*, and survival monitored twice daily over a period of 7–14 days. For neutrophil depletion experiments, mice were injected intraperitoneally with 0.5 mg of anti-Ly6G depleting antibody (clone 1A8, BioXcell) or 0.5 mg of control rat IgG antibody (clone 2A3, BioXcell) 24 h before *B. anthracis* infection. For CFU determination, animals were euthanized 24 h post-infection. Liver, spleen, and kidney samples were homogenized in PBS. Blood and organ samples were then serial diluted in PBS and CFU recovered was enumerated on BHI agar plates.

### Splenocyte staining and flow cytometry

Single cell splenocytes were prepared by meshing the spleen on a 40 μM strainer using the rubber end of the plunger. Red blood cell lysis was accomplished using lysis buffer (eBioscience), and splenocytes stained in FACS buffer (PBS+0.5% BSA) for 20 min before analysis by FACS Calibur (BD Biosciences). The fluorescent conjugated antibodies CD11b (Clone M1/70, BD Biosciences) and Ly6G (Clone 1A8, Biolegend) were used to identify the infiltrating population in the mouse spleen. Staining specificity was verified using corresponding isotype controls.

### Mouse serum cytokine levels

Mice were euthanized as described above and blood was collected via cardiac puncture. Serum was separated from whole blood using Microtainer serum separator tubes (BD Biosciences) by spinning clotted whole blood at 10,000 × g for 2 min. Serum concentration of IFNγ, TNF, and KC was determined using the Cytometric Bead Array Flex kit (BD Biosciences). Capture beads and undiluted serum samples were mixed and applied to a 96-well 1.2 μm Multiscreen Filter plate (EMD Millipore), then incubated at room temperature for 1 h. Detection beads were added to the filter plate and incubated for an additional 1 h. After washing with wash buffer, the beads were transferred to a 96-well round bottom plate and analyzed on a FACSCanto II flow cytometer (BD Biosciences). Quantification of data was performed using the FCAP Array software (BD Biosciences).

### Neutrophil killing assay

Freshly isolated human neutrophils were resuspended in RPMI+2% FBS and apportioned at 2 × 10^5^ cells/well in a 96-well plate. *B. anthracis* strains at OD_600nm_ = 0.4 were added to each well at an approximate MOI of 5:1 bacteria to neutrophils. Plates were spun for 5 min at 290 × g to pull bacteria down onto neutrophils, then incubated for 15 min at 37°C with 5% CO_2_. Bacteria survival was calculated as percent bacteria recovered compared to the inoculum. For pharmacological rescue studies of *B. anthracis* killing, neutrophils were treated with 10 kIU/ml aprotinin, 10 μg/ml cytochalasin D, or 10 mM N-acetyl-L-cysteine, alone or in combination, for 30 min before addition of bacteria.

### Antimicrobial peptide release

Neutrophils were infected with *B. anthracis* at an approximate MOI of 1 as described above. After 1 h of infection, plates were spun down at 290 × g for 5 min and supernatants dotted on nitrocellulose membranes (BioRad) and allowed to air dry. Membranes were blocked in PBS blocking buffer (Li-Cor) for 1 h, then incubated overnight with 1:1,000 dilution anti–LL37 (Santa Cruz Biotech) or anti α-defensin (Santa Cruz Biotech). Membranes were then washed with 0.1% PBS-Tween and incubated for 1 h with goat anti-mouse secondary antibody (Li-Cor). After washing with 0.1% PBS-Tween, membranes were imaged on Li-Cor Odyssey CLx. Densitometry analysis was performed using ImageJ software (National Institutes of Health). For elastase activity assay, supernatants from infected neutrophils were collected as described above. Supernatants were incubated with 1 mM of the colorimetric elastase substrate N-methoxysuccinyl-Ala-Ala-Pro-Val-p-nitroanilide (p-nitroanilide) (Sigma). Elastase activity was measured after 30 min on a PerkinElmer EnSpire Alpha plate reader at an optical density of 405 nm (OD_405_).

### ROS generation assay

Neutrophils in HBSS+/+ were incubated for 20 min with 20 μM 2′,7′-dichlorofluorescein (DCFH-DA) (Sigma) at 37°C with continuous gentle mixing. Cells were spun down at 300 × g for 10 min, then resuspended in HBSS+/+ to final concentration = 2 × 10^6^ cells/ml; a 100 μl volume of cells were added to each well of a 96-wells plate. Next an MOI of approximately 5:1 *B. anthracis* was added to the neutrophils, and 25 nM phorbol myristate acetate (PMA) used as a positive control. The plate was spun at 300 × g for 5 min and incubated at 37°C with 5% CO_2_. Fluorescent signal was read at excitation 485 nm/emission 530 nm on a SpectraMAX Gemini EM fluorescence reader at 30 min intervals over 180 min.

### Transwell chemotaxis

*B. anthracis* (2–3 × 10^6^ CFU) in HBSS+/+ were placed in the lower well of a 3.0 μm polycarbonate Transwell (Corning), and the plate centrifuged for 5 min at 200 × g. Transwell inserts were then gently placed inside the wells, and 1 × 10^6^ neutrophils in HBSS+/+ are placed inside each insert. As a positive control, 100 nM N-formylmethionyl-leucyl-phenylalanine (fMLP) (Sigma) was added to lower wells, while HBSS+/+ media in the lower well alone served as a negative control. Transwells were incubated at 37°C, 5% CO_2_ for 45 min, after which the insert was gently removed. The content of the lower well was collected and the total number of neutrophils determined by running the samples on a FACS Calibur (BD Bioscience) flow cytometer with data analysis by FlowJo v. 9.7.7 (Tree Star, Inc).

### NET production and staining

NET production was performed as previously described (Von Köckritz-Blickwede et al., 2010). Briefly, 2 × 10^5^ neutrophils in HBSS+/+ were seeded in a 96-wells plates, and 25 nM PMA (positive control) or *B. anthracis* at approximately an MOI of 5 were added to the wells. The plate was spun down at 290 × g for 5 min and incubated at 37°C, 5% CO_2_ for 4 h. Next, 500 U/ml of micrococcal nuclease (Sigma) was added to the wells and incubated at room temperature to digest the NETs. Then 5 mM EDTA was added to stop the activity of the micrococcal nuclease and cells removed by centrifugation. Supernatant was collected and incubated with Quanti-iT PicoGreen for 5 min at room temperature. Fluorescence signal was measured at excitation 480 nm/emission 520 nm on a SpectraMax Gemini EM fluorescence reader. To visualize NET structures, 5 × 10^4^ neutrophils in HBSS+/+ were seeded in borosilicate 8-well chamber slides (Nunc). Next 25 nM PMA (positive control) or 25 × 10^4^ CFU of *B. anthracis* in HBSS+/+ were added to each chamber. Slides were then incubated at 37°C, 5% CO_2_ for 4 h, after which 4% paraformaldehyde was added and the content of the chambers fixed for 30 min at room temperature. Chambers were then washed three times with PBS, and NET structures stained with 5 μM SYTOX Green (Invitrogen). NETs were imaged on a Zeiss Observer D1 inverted fluorescent microscope.

### Neutrophil phagocytosis

Neutrophils (2 × 10^5^) in RPMI+2% FBS were added to 96-well plates and infected with GFP-expressing *B. anthracis* strains at an approximate MOI of 2:1. To control for bacterial adhesion to neutrophils, 10 μg/ml of cytochalasin D (Sigma) was added prior to infection and incubated at 37°C, 5% CO_2_ for 20 min. Uninfected neutrophils were used as negative controls. The plate was centrifuged at 290 × g for 5 min and incubated at 37°C with 5% CO_2_ for 30 min. Cells were then washed three times with PBS, resuspended in 0.4% trypan blue, and analyzed on a FACS Calibur (BD Bioscience). The percentage of fluorescent cells was determined based on the distribution of cytochalasin D treated controls. Data from the flow cytometer was analyzed using FlowJo v. 9.7.7 (Tree Star, Inc).

### Neutrophil viability

Neutrophils (2 × 10^5^) in RPMI+2% FBS were added to 96-well plates and infected with *B. anthracis* strains at an approximate MOI of 1. The plate was centrifuged at 290 × g for 5 min and incubated at 37°C, 5% CO_2_ for 15 min. Uninfected neutrophils served as a negative control. Propidium iodide (Invitrogen) was added to stain for dead cells, and samples analyzed on a FACS Calibur (BD Bioscience) flow cytometer using FlowJo v. 9.7.7 (Tree Star, Inc). To determine whether cell death was associated with phagocytosis of bacteria, neutrophils were infected with GFP expressing anthrax strains as described before. As a control, neutrophils were treated with 10 μg/ml of cytochalasin D for 20 min before infection to inhibit phagocytosis; then 15 min after infection, neutrophils were stained with propidium iodide and run on FACS Calibur as described above.

### Statistical analysis

At least three independent experiments were performed for all data shown, and neutrophils from at least three different healthy volunteers were used for experiments. Differences between experimental groups were analyzed by Student *t*-test and ANOVA. Difference in animal survival following infection was analyzed using log-rank test. Results are shown as mean ±SEM. A *p-value* < 0.05 was considered statistically significant.

## Results

### Neutrophil depletion precipitates death in a mouse model of systemic infection

**Figure 1:**
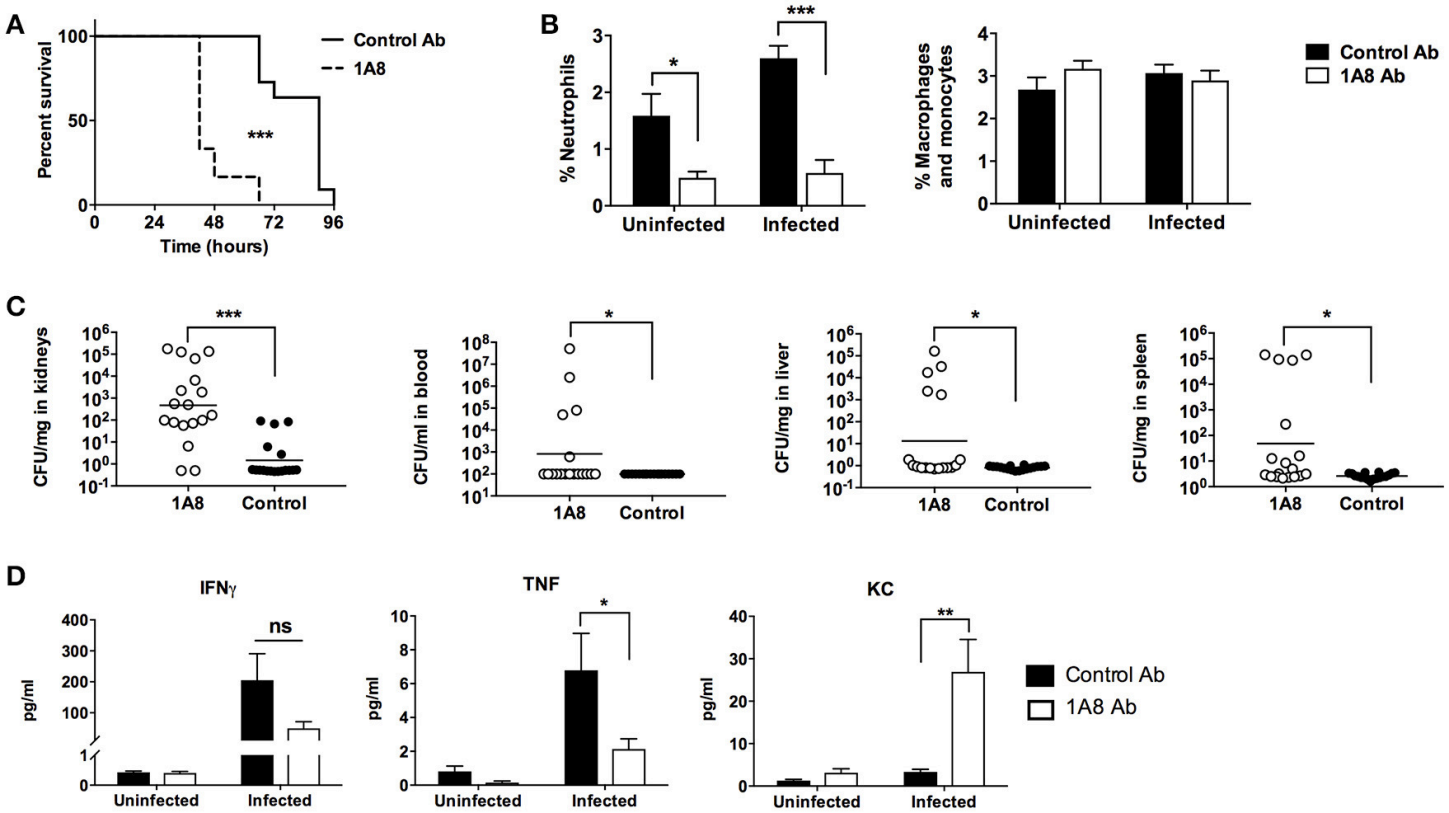
*B. anthracis* Sterne infection of neutrophil-depleted mice. **(A)** Mice treated with the neutrophil-depleting 1A8 antibody show increased mortality upon *B. anthracis* challenge compared to control antibody-treated animals. 1A8 antibody treated, *n* = 12; Control antibody treated, *n* = 11. **(B)** Splenocytes from depleting antibody treated and control antibody treated animals were stained with CD11b and Ly6G antibody, where percentage neutrophil is defined as CD11b+, Ly6G+ cells/all splenocytes, and percentage macrophage/monocytes are defined as CD11b+, Ly6G− cells/all splenocytes. Infected 1A8 antibody treated and infected control antibody treated, *n* = 18/group; uninfected 1A8 antibody treated and uninfected control antibody treated, *n* = 6/group. **(C)** Bacteria recovered from organs (CFU/mg) and blood (CFU/ml) of 1A8 antibody and control antibody treated infected mice. **(D)** Serum cytokine levels of TNF, IFNγ, and KC from 1A8 antibody treated and control antibody treated mice. ^*^P < 0.05, ^**^P < 0.01, ^***^ *P* < 0.001.

Neutrophils are required for maximal survival of mice following aerosolized *B. anthracis* spores challenge (Cote et al., 2006), and human neutrophils can kill both *B. anthracis* spores and vegetative bodies (Mayer-Scholl et al., 2005). We sought to determine whether neutrophils play a protective role in a mouse systemic infection model using the metabolically active vegetative form of *B. anthracis* Sterne. C57BL/6 mice were injected with 1A8 antibody to selectively deplete neutrophils or an isotype control antibody (Daley et al., 2008). Animals were then infected via intraperitoneal injection and mortality was monitored twice daily. Splenic cell counts confirmed that 1A8 treatment depleted neutrophils while preserving monocyte/macrophages in both uninfected and infected animals (Supplementary Figure S1). WT *B. anthracis* challenge caused a sharp rise in mortality at 48 h post-infection in the 1A8 antibody-treated group, with all animals succumbing by 72 h (Figure 1A). In contrast, only one third of the control antibody-treated animals had died by 72 h post-infection (Figure 1A). Examination of splenic leukocyte populations confirmed a significant reduction in the percentage of neutrophils in 1A8 antibody-treated animals compared to the control group, whereas the respective percentage of macrophage/monocytes did not differ significantly (Figure 1B). Consistent with the mortality data, significantly higher CFU counts of *B. anthracis* were recovered from the liver, spleen, blood, and most notably, the kidneys of 1A8 antibody-treated neutropenic mice compared to control animals (Figure 1C). Serum concentrations of TNF and IFNγ, which are known to be secreted by neutrophils (Tecchio et al., 2014), were diminished in 1A8-treated mice (Figure 1D). Conversely, significantly higher levels of the neutrophil chemokine KC were detected in the neutrophil-depleted mice, an effect that may be mediated by a negative feedback loop (see discussion) (Figure 1D).

### Key neutrophil functions are not modulated by endogenous anthrax toxin expression

The specific mechanism(s) by which neutrophil contribute to restricting *B. anthracis* dissemination are unclear, though it has been suggested that release of the antimicrobial peptide α-defensin and/or the process of autophagy may each play a role in neutrophil-mediated anthrax killing (Mayer-Scholl et al., 2005; Ramachandran et al., 2015). *In vitro*, pharmacological administration of purified lethal toxin or edema toxin have been reported to inhibit certain neutrophil functions, including chemotaxis, phagocytosis, and ROS generation (O'brien et al., 1985; Crawford et al., 2006; Szarowicz et al., 2009; Weiner et al., 2014).

**Figure 2:**
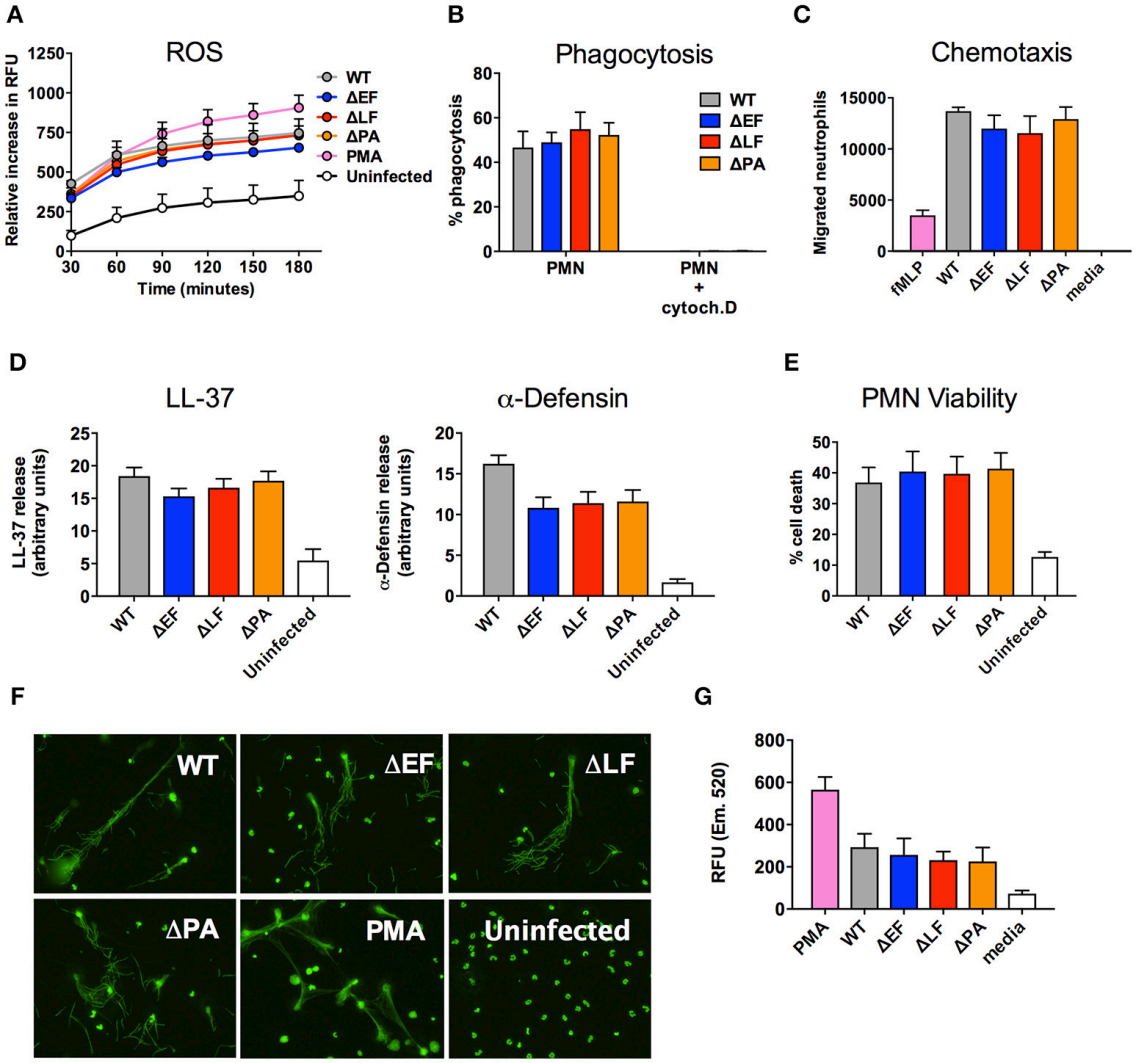
Neutrophil functions during *B. anthracis* infection. **(A)** Generation of ROS by neutrophils infected with the indicated strains of *B. anthracis*. **(B)** Neutrophil phagocytosis of GFP-expressing *B. anthracis* strains treated in the presence or absence of cytochalasin D. **(C)** Representative data of neutrophil migration toward *B. anthracis* strains through a Transwell system. **(D)** Densitometry analysis of LL-37 and alpha-defensin dot blots from supernatant of neutrophils infected with the indicated strain of *B. anthracis*. **(E)** Neutrophil viability following *B. anthracis* infection as determined by PI staining. **(F)** Representative image of SYTOX Green-stained NETs following infected of neutrophils with the indicated *B. anthracis* strain. **(G)** Quantification of NET released by neutrophils following *B. anthracis* infection. Unless otherwise stated, data shown are from a combination of at least three independent experiments.

To determine whether endogenous expression of anthrax toxins inhibited neutrophil function during live infection with vegetative cells, we employed WT *B. anthracis* and isogenic strains with genetic deletion of lethal factor (ΔLF), edema factor (ΔEF), or protective antigen (ΔPA); expression of the expected toxin profile in the strains has been confirmed by RT-PCR and function assays (Guichard et al., 2010; Ali et al., 2011). The effects of endogenous anthrax toxin expression on the ability of neutrophils to generate ROS, phagocytose bacteria, release antimicrobial peptides and migrate during infection, as well as the impact of toxin expression on neutrophil survival, were evaluated (Figure 2). ROS expression by freshly isolated neutrophils was measured by the fluorescent signal of the indicator dye DCFH-DA, with PMA, a potent stimulus of neutrophil activation, serving as a positive control. Neutrophils quickly generated ROS following contact with *B. anthracis* bacteria, and produced similar levels of ROS in response to WT and toxin mutant strains of the bacteria (Figure 2A). Another key antimicrobial function of neutrophil is the cell's ability to rapidly engulf bacteria. Efficient neutrophil phagocytosis of GFP-expressing WT *B. anthracis* was noted and similar rates of phagocytosis of isogenic toxin mutants was noted (Figure 2B). As a negative control to rule out GFP signal from simple attachment, we detected little to no GFP signal from neutrophils pretreated with cytochalasin D to inhibit actin polymerization required for phagocytic engulfment (Figure 2B).

Prior studies have shown that pretreatment of neutrophils with purified lethal toxin and edema toxin can inhibit neutrophil actin polymerization (During et al., 2005; Szarowicz et al., 2009), and that injection of lethal toxin in mice reduced neutrophil migration following localized PMA stimulation (Weiner et al., 2014). We assessed using a Transwell assay if non-toxin-pretreated neutrophils migrated toward live *B. anthracis* vegetative bodies, and whether toxin expression by the bacterial cells would affect the degree or rate of neutrophil chemotaxis. We found that live *B. anthracis* bacteria are a potent chemoattractant, attracting approximately three times more neutrophils than the well-studied bacterial-derived chemotactic factor, fMLP (Figure 2C). However, expression of anthrax toxins did not affect neutrophil migration in this context, as all three mutant strains of anthrax (ΔEF, ΔLF, and ΔPA) stimulated equal neutrophil recruitment compared to the WT parent strain (Figure 2C).

Ketotifen, a compound that inhibits mast cell degranulation, has been shown to reduce *B. anthracis* LT-induced vascular leakage (Gozes et al., 2006). However, it is not known whether anthrax infection itself induces neutrophil degranulation or whether anthrax toxins modulate release of key antimicrobial peptides contained within these granules. Human cathelicidin LL-37 and alpha-defensins are small cationic peptides found in neutrophil specific granules and azurophilic granules, respectively (Dewald et al., 1975; Rice et al., 1987; Cowland et al., 1995). Neutrophils incubated with our panel of *B. anthracis* strains for 1 h were stimulated to release LL-37 and alpha-defensin as measured by dot blot (Supplementary Figure S2A), while elastase release was quantified using an elastase enzymatic activity assay (Supplementary Figure S2B). No statistically significant differences between WT and toxin-deficient mutant bacteria were found when examining the blot intensity of LL-37 and alpha-defensin (Figure 2D). The same was observed for elastase release, as similar elastase activity was found in the supernatant of all neutrophils infected by anthrax, regardless of strain (Supplementary Figure S2B).

LT induces apoptosis in macrophages, however purified LT and ET do not significantly affect the viability of neutrophils (Crawford et al., 2006; Szarowicz et al., 2009). However, the influence of live anthrax bacteria on neutrophil viability has not been assessed. We infected neutrophils with the five anthrax strains and assessed cell viability with propidium idodine staining after 15 min. While strains of anthrax induced neutrophil cell death following infection, comparison among mutants indicated that the genetic presence or absence of anthrax toxin was not a significant contributor to this reduced viability (Figure 2E). Neutrophil cell death can be induced by ROS (Coxon et al., 1996) and phagocytosis (Kobayashi et al., 2003), two processes that we observed during anthrax infection (Figures 2A,B). Given that cytochalasin D treatment effectively blocked neutrophil cell death (Supplementary Figure S3), we wanted to determine the contribution of phagocytosis to neutrophil death during anthrax infection. We infected neutrophils with GFP expressing anthrax strains and stained the cells with propidium iodide to assess viability. We found that between 40 and 50% of propidium iodide positive neutrophils are associated with GFP-expressing bacteria after 15 min, and like in Figure 2E, the absence or presence of anthrax toxin did not significantly influence the percentage of bacteria associated with dead cells (Supplementary Figure S3).

NET formation is an important host defense against a wide range of pathogens, including *Candida albicans, Staphylococcus aureus*, and *Listeria monocytogenes* (Brinkmann et al., 2004; Urban et al., 2006). NETs are released upon neutrophil activation and consist of a fibrous matrix of DNA and antimicrobial proteins that ensnare and kill bacteria. We infected neutrophils with the four strains of anthrax in borosilicate chamber slides, then fixed and stained for NETs using SYTOX Green stain; PMA was used as a positive control for NET release. We found that all four strains of anthrax induced structures characteristic of NETs, and observed bacilli associated with these long, fibrous structure (Figure 2F). Quantification of extracellular DNA using the Quant-iT Pico Green kit indicated that similar to other neutrophil functions, expression of anthrax toxins did not affect the level of neutrophil NET release (Figure 2G).

In summary, exposure to anthrax bacteria rapidly induced several neutrophil activation phenotypes, including chemotaxis, phagocytosis, ROS production, degranulation and release of cationic antimicrobial peptides, and NET formation. Endogenous expression of anthrax toxins (LT or ET) by the living anthrax bacteria was insufficient, either in quantity or kinetics, to significantly inactivate or alter these crucial host defense mechanisms in the *ex vivo* model systems tested.

### Neutrophils utilize multiple mechanisms to kill *B. anthracis*

**Figure 3:**
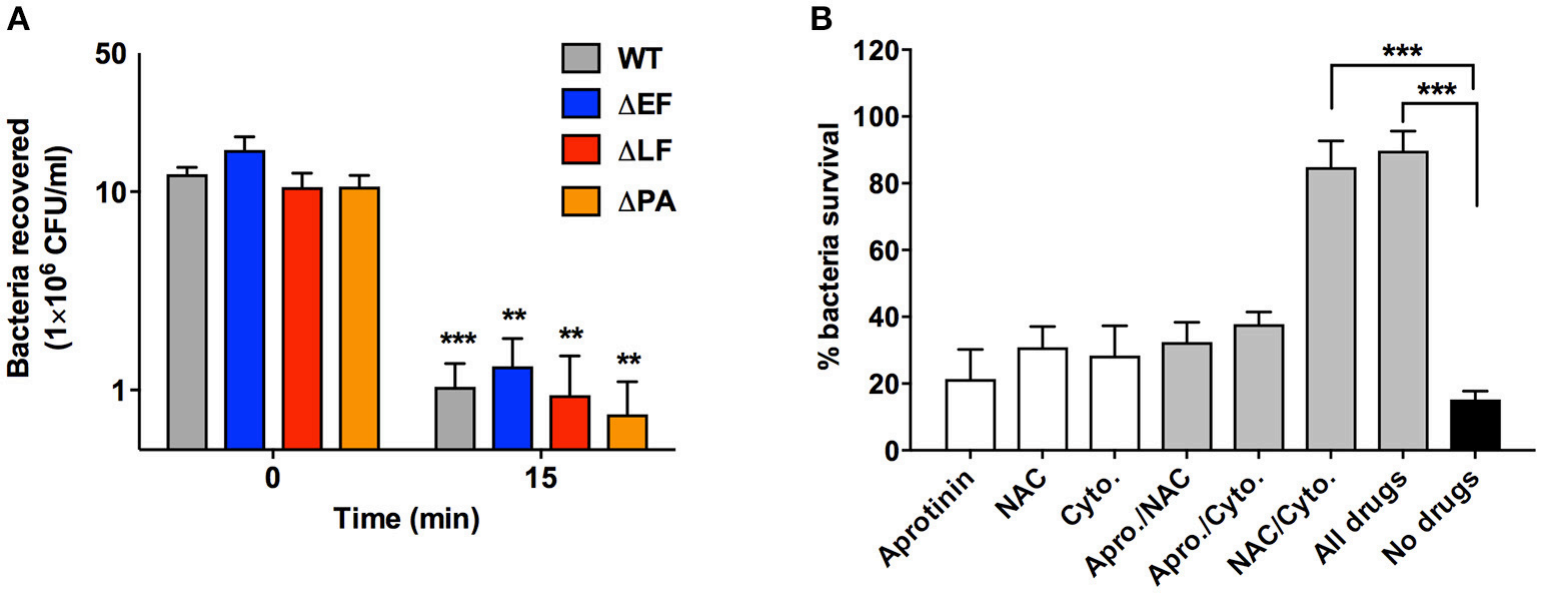
Mechanism of neutrophil anthrax killing. **(A)** CFU/ml of *B. anthracis* strains recovered at time 0 and 15 min after neutrophil infection. **(B)** Neutrophils were pretreated with the indicated compound for 30 min, then infected with wild type *B. anthracis* Sterne and the percentage of bacterial survival enumerated. Percentage bacterial survival is calculated by dividing the CFU/ml of bacteria recovered in the presence of neutrophils over the CFU/ml of bacteria recovered in the absence of neutrophils. ^**^P < 0.01 and ^***^P < 0.001. All data shown are from a combination of at least three independent experiments.

We next sought to determine whether individual anthrax toxins contribute to anthrax resistance or sensitivity to neutrophil killing. We infected neutrophils with WT *B. anthracis* or the isogenic mutant strains ΔEF, ΔLF, or ΔPA, then enumerated the bacteria recovered after 15 min. We observed an approximately 1 log-fold reduction in *B. anthracis* bacteria within this infection period, but this effect was largely toxin independent as deletion of individual toxins did not accelerate nor decrease the rate of neutrophil killing of the bacteria (Figure 3A).

We next probed how certain key antimicrobial mechanisms utilized by neutrophils, specifically phagocytosis, ROS production and cathelicidin antimicrobial peptide, might contribute to the observed rapid elimination of anthrax bacteria by employing a series of specific pharmacological inhibitors. Actin polymerization inhibitor cytochalasin D was used to evaluated the role of phagocytosis. To block ROS-mediated killing, we used the ROS scavenger N-acetylcysteine (NAC). We chose NAC over the frequently studied NADPH oxidase inhibitor DPI, previously shown unable to rescue anthrax from neutrophil killing (Mayer-Scholl et al., 2005), to avoid potential confounding effects from DPI-induced apoptosis in human neutrophil-like cells (Li et al., 2003), since NAC actually protects neutrophils against apoptosis (Watson et al., 1996). *B. anthracis* is also susceptible to killing by cathelicidin antimicrobial peptides (Mcgillivray et al., 2009, 2012) produced in abundance by neutrophils, and maturation of the human cathelicidin LL-37 can be blocked using the proteinase 3 inhibitor aprotinin (Sørensen et al., 2001). These inhibitors were tested individually and in combinations to determine whether 30 min pretreatment would affect different neutrophil functions that may synergize to kill *B. anthracis*. After 15 min of infection, we found that approximately 70–80% of the bacterial inoculum was killed by untreated neutrophils or those treated with only a single compound (Figure 3B). The dual combination of aprotinin/NAC and aprotinin/cytochalasin D also did not significantly reduce bacterial killing. However, the combination of NAC/cytochalasin D and a combination of all three inhibitors provide a ~90% rescue of *B. anthracis* from neutrophil killing (Figure 3B). As a control, we showed that the inhibitors did not directly contribute to bacteria killing, as anthrax incubated with inhibitors in the absence of neutrophils exhibited no decrease in the number of viable CFUs (Supplementary Figure S4). These results indicate that phagocytosis and ROS production are key effectors of anthrax killing by human neutrophils.

## Discussion

Despite playing a frontline role in the control and clearance of bacterial infections, neutrophils have been more difficult to study in comparison to other innate immune cells such as macrophages due to their short lifespan and high reactivity. The interaction between neutrophils and anthrax has particularly been understudied. Previous *in vitro* studies on the effect of purified anthrax toxins have shown the potential for higher level toxin exposure to inhibit neutrophil chemotaxis, ROS release, and phagocytosis, suggesting that this strategy might be employed by the pathogen to weaken the immune response (O'brien et al., 1985; Crawford et al., 2006; Szarowicz et al., 2009; Weiner et al., 2014). However, in our *ex vivo* studies with freshly purified human neutrophils and WT and anthrax toxin mutant strains we found that, contrary to the findings with purified toxins, the presence or absence of anthrax toxins does not have any significant effect on key neutrophil functions. The absence of an inhibitory effect of anthrax toxin on neutrophils may be attributed to the rapid killing of anthrax by neutrophils, wherein approximately 80–90% of the initial bacteria inoculum is eliminated within 15 min of co-incubation, which likely outpaces the kinetics of endogenous production of anthrax toxin and their ability to target cellular signaling pathways.

Efficient killing of anthrax makes neutrophils an important line of defense against the pathogen during infection. In our study we found that depletion of neutrophils with 1A8 antibody lead to enhanced mortality and increased bacterial burden in the organs and blood compared to mice administered a control antibody. Significantly higher levels of murine neutrophil chemokine KC was found in the serum of neutrophil-depleted animals. This phenotype had previously been observed in neutrophil-depleted mice following surgical injury or transgenic mice that lack the functional CXCR2 receptor to engage KC (Kielian et al., 2001; Endlich et al., 2002), and may reflect a lack of an inhibitory feedback signal from infiltrating neutrophils, leading to persistent KC expression. In contrast to KC, both TNF and IFNγ were diminished in neutrophil-depleted animals. Immunomodulatory effects of TNF and IFNγ include the recruitment of dendritic cells, regulation and differentiation of macrophages and T cells. Thus, in addition to controlling anthrax dissemination, neutrophils may have downstream effects to support the activity and function of other immune cells during anthrax infection (Nathan, 2006).

The importance of neutrophils during anthrax infection is closely tied to their effective killing of the pathogen. Both our study and work by other groups have shown that neutrophils phagocytose and kill *B. anthracis* spores and vegetative bodies (Mayer-Scholl et al., 2005). Alpha-defensins and autophagy have been indicated to play contributory role in neutrophil killing of anthrax, and ROS inhibition alone does not rescue anthrax from killing (Mayer-Scholl et al., 2005; Ramachandran et al., 2015). In our study, we similarly found that scavenging of ROS alone was insufficient to block neutrophil anthrax killing. *B. anthracis* produces bacterial-derived nitric oxide (NO), which is used to counteract the hydroxyl radicals generated by Fenton effect as a defense against phagocyte oxidative burst killing (Shatalin et al., 2008). However, we were able to rescue *B. anthracis* from neutrophil killing by inhibiting phagocytosis and ROS through the combination of cytochalasin D (to block phagocytosis) and ROS scavenger NAC. This indicates that while neutrophil-derived ROS alone is unable to exert bactericidal effect against anthrax, it is able to synergize with other antimicrobial functions of neutrophils to promote intracellular killing in the phagolysosome. One of the consequences of neutrophil phagocytosis is a form of apoptosis termed phagotycosis-induced cell death (PICD), which can be triggered by the ingestion of a variety of bacteria (DeLeo, 2004; Kobayashi and DeLeo, 2009). PICD is a pathway that leads to the resolution of the inflammatory response through the process of efferocytosis. We found that close to half of all dead neutrophils were associated with anthrax bacteria, suggesting that PICD may be an important mechanism in anthrax clearance and contribute to termination of the inflammatory response post-infection. We also show for the first time that anthrax induces NET formation by neutrophils. The development of NETs *ex vivo* takes longer to proceed (1–4 h) to account for the hyper-acute killing in our human neutrophil killing assays (seen within 15–30 min), but can also be a possible immune mechanism to further limit the dissemination of bacteria after activated neutrophils have undergone this specialized cell death process *in vivo* (Brinkmann and Zychlinsky, 2007).

We conclude that neutrophil killing of anthrax is mediated through a combination of different antimicrobial processes acting in concert, and that the relative imperviousness of these rapidly acting phagocytic cells to anthrax toxin-mediated functional impairment makes them an effective and essential first line of defense against the pathogen.

## Author contributions

JL and VN designed the study. JL and SA performed the experiments. JL, SA, EB, and VN analyzed data. JL and VN wrote the manuscript.

### Conflict of interest statement

The authors declare that the research was conducted in the absence of any commercial or financial relationships that could be construed as a potential conflict of interest.
