# Supplementary material for: Innate Immune Interactions between Bacillus anthracis and Host Neutrophils
Source: Front Cell Infect Microbiol. 2018 Jan 22;8:2. doi: 10.3389/fcimb.2018.00002 (PMC5786542; doi:10.3389/fcimb.2018.00002)
Supplement: Supplementary file 1 [file DataSheet1.docx]

***Supplementary Material***

**Innate immune interactions between *Bacillus anthracis* and host neutrophils**

*Janet Z. Liu^1^, Syed Raza Ali^1^, Ethan Bier^2^, Victor Nizet^1,3*^*

*^1^Department of Pediatrics, Division of Host-Microbe Systems and Therapeutics, UC San Diego, La Jolla, CA, USA*

*^2^Section of Cell and Developmental Biology, Division of Biological Sciences, UC San Diego, La Jolla, CA, USA*

*^3^Skaggs School of Pharmacy and Pharmaceutical Sciences, UC San Diego, La Jolla, CA, USA*

*Corresponding author

Victor Nizet: email [vnizet@ucsd.edu](mailto:vnizet@ucsd.edu)


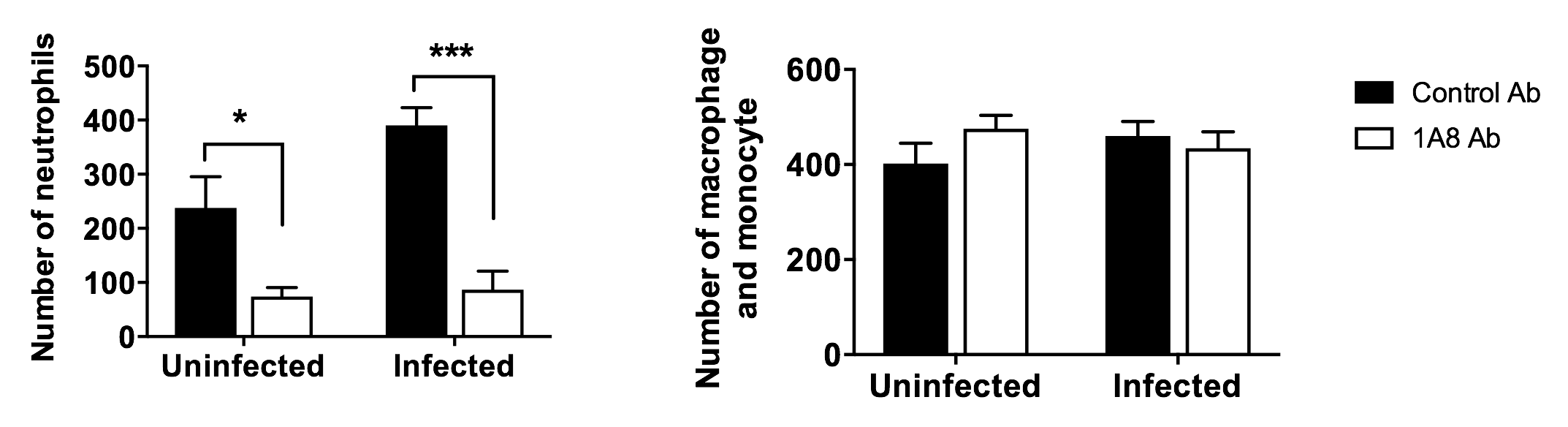


**Supplementary Figure S1 | Total number of neutrophil, macrophage and monocytes from murine spleen.** Number of neutrophil and macrophage/monocytes recovered from the spleen of uninfected and wild-type *Bacillus anthracis* Sterne infected mice, treated with control antibody (Ab) or with neutrophil depleting 1A8 antibody.

* P< 0.05, *** P< 0.001


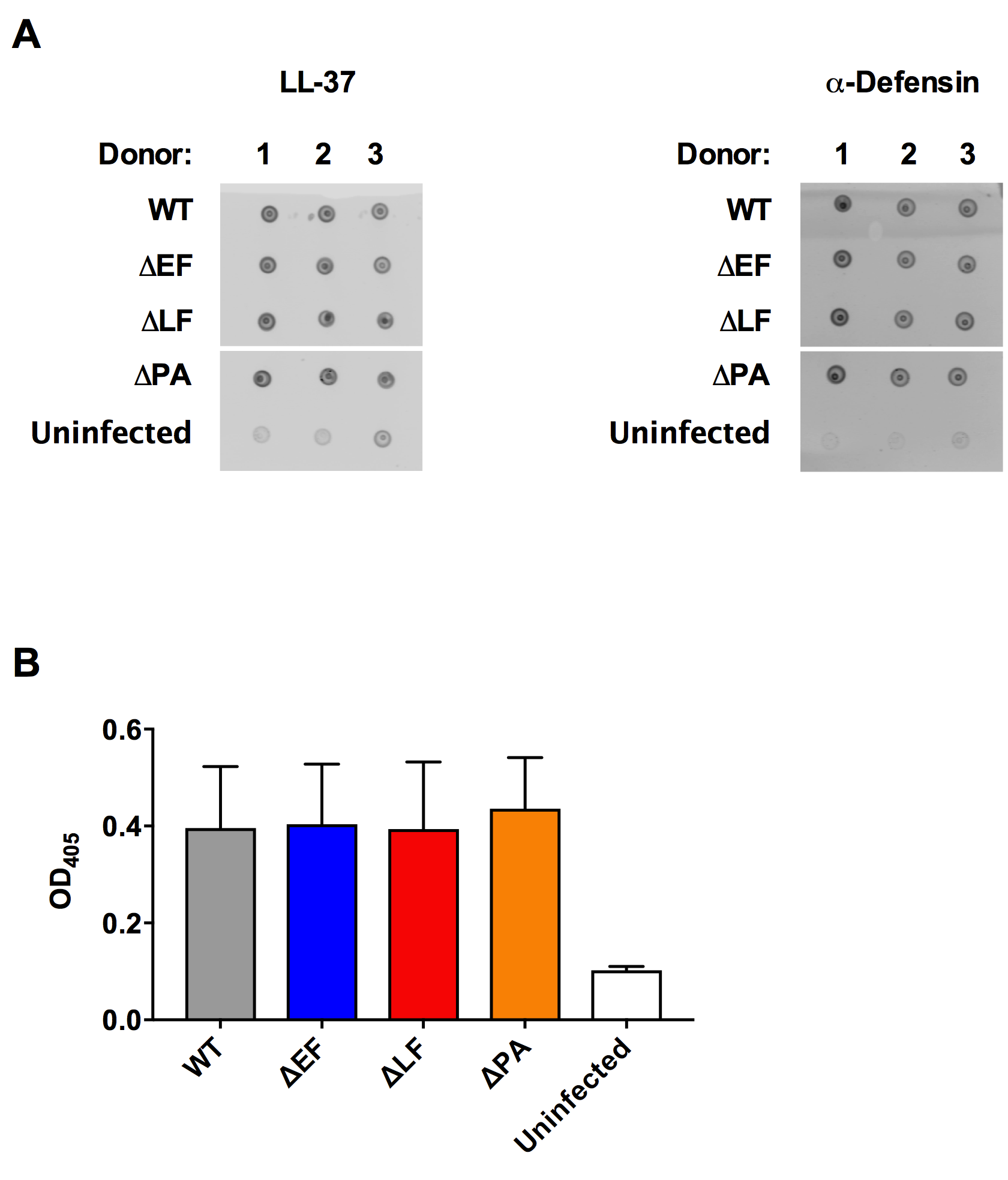


**Supplementary Figure S2 | Release of neutrophil granular proteins.** (A) Dot-blots of LL-37 and α-defensin released by neutrophils infected the indicted *Bacillus anthracis* Sterne strain. Supernatant from infected neutrophils from three donors were collected 1 h post-infection and dotted on nitrocellulose membrane, then incubated with antibody for the indicated antimicrobial proteins. (B) Elastase assay as a read out of neutrophil elastase release during anthrax infection. Supernatant from infected neutrophils were collected at 1 h post-infection and mixed with p-nitroanilide. The resulting signal for elastase activity was measured using a spectrophotometer.


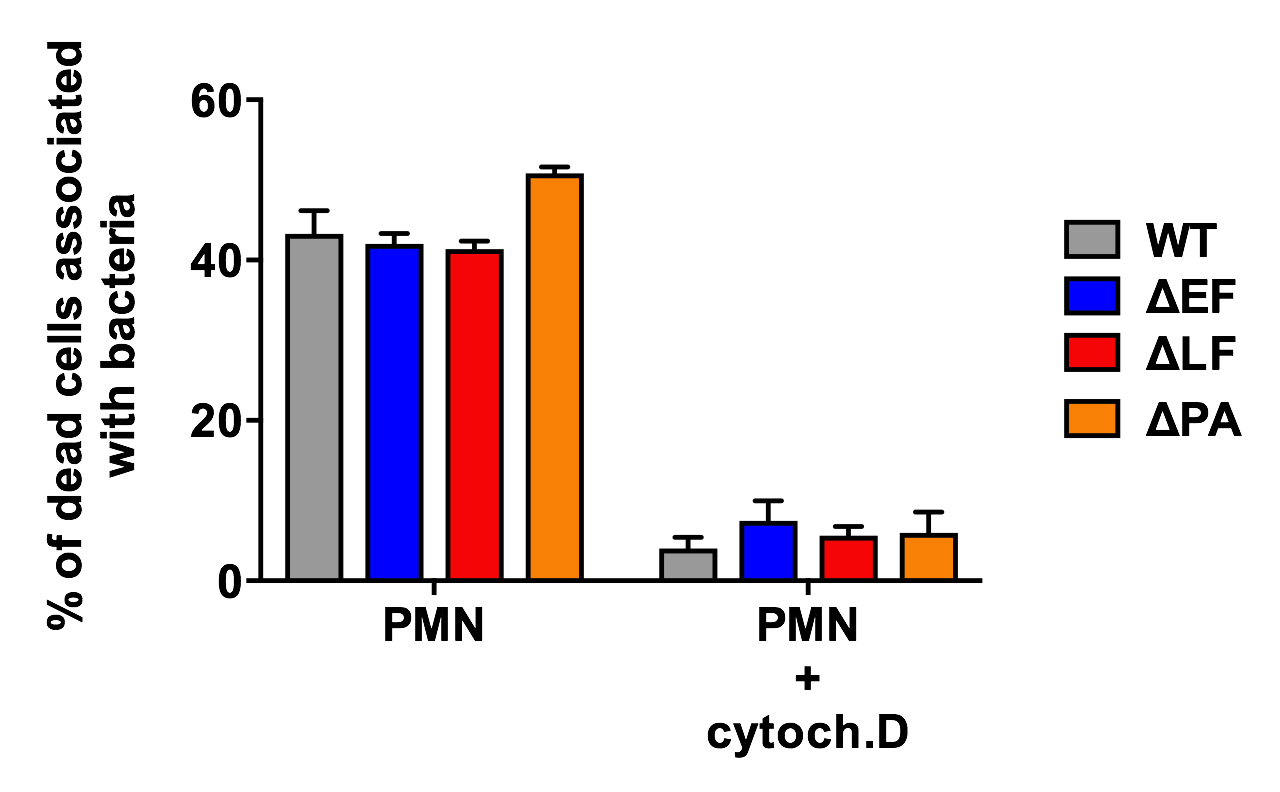


**Supplementary Figure S3 | Percent of dead neutrophils associated with bacteria.** Neutrophils were infected with the indicated strain of GFP-expressing *Bacillus anthracis* Sterne and stained 15 min post-infection with propidium iodide. As a control, neutrophils were incubated for 20 min before infection with cytochalasin D to inhibit phagocytosis.


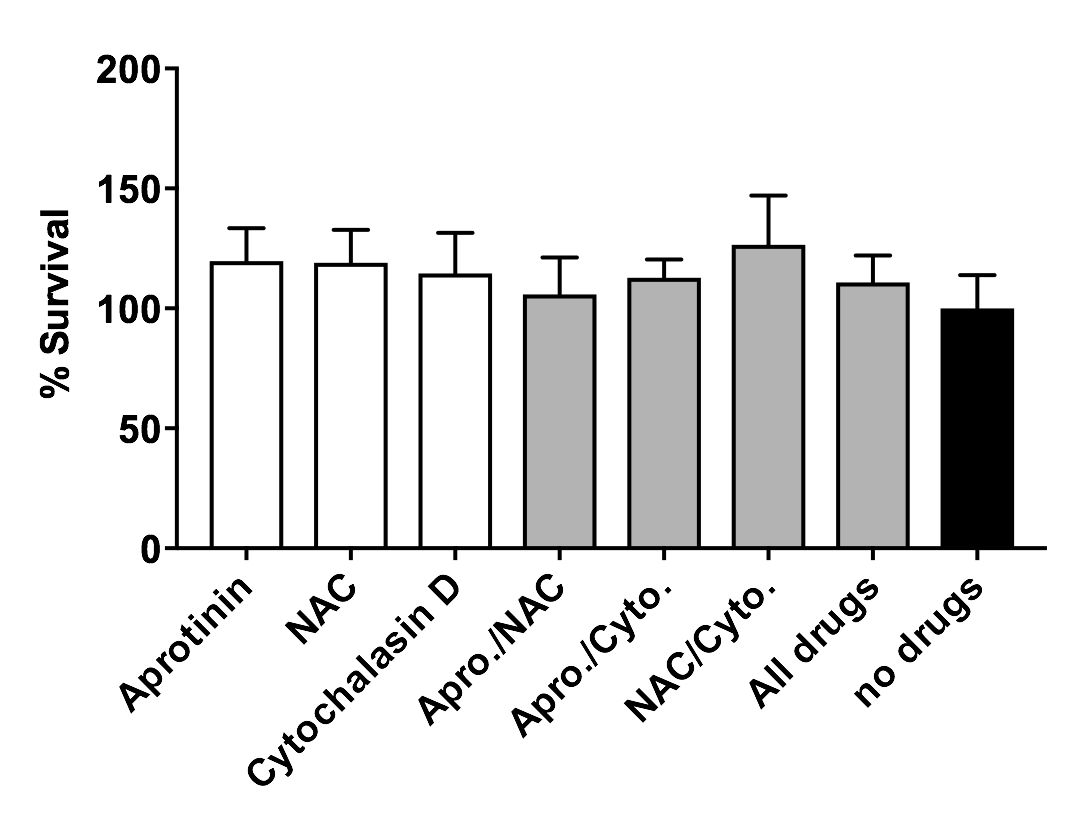


**Supplementary Figure S4 | Growth of wild-type anthrax in the presence of neutrophil function inhibitors.** Wild-type *Bacillus anthracis* Sterne was incubated for 15 min in the presence of the indicated neutrophil function inhibitors. The percent survival was determined by plating on BHI agar and normalized to untreated anthrax.
